# Supplementary material for: A candidate multimodal functional genetic network for thermal adaptation
Source: PeerJ. 2014 Sep 30;2:e578. doi: 10.7717/peerj.578 (PMC4183952; doi:10.7717/peerj.578)
Supplement: Supplemental Information 1 — Yellow boxes denote research resources provided in this paper. [file peerj-02-578-s001.docx]

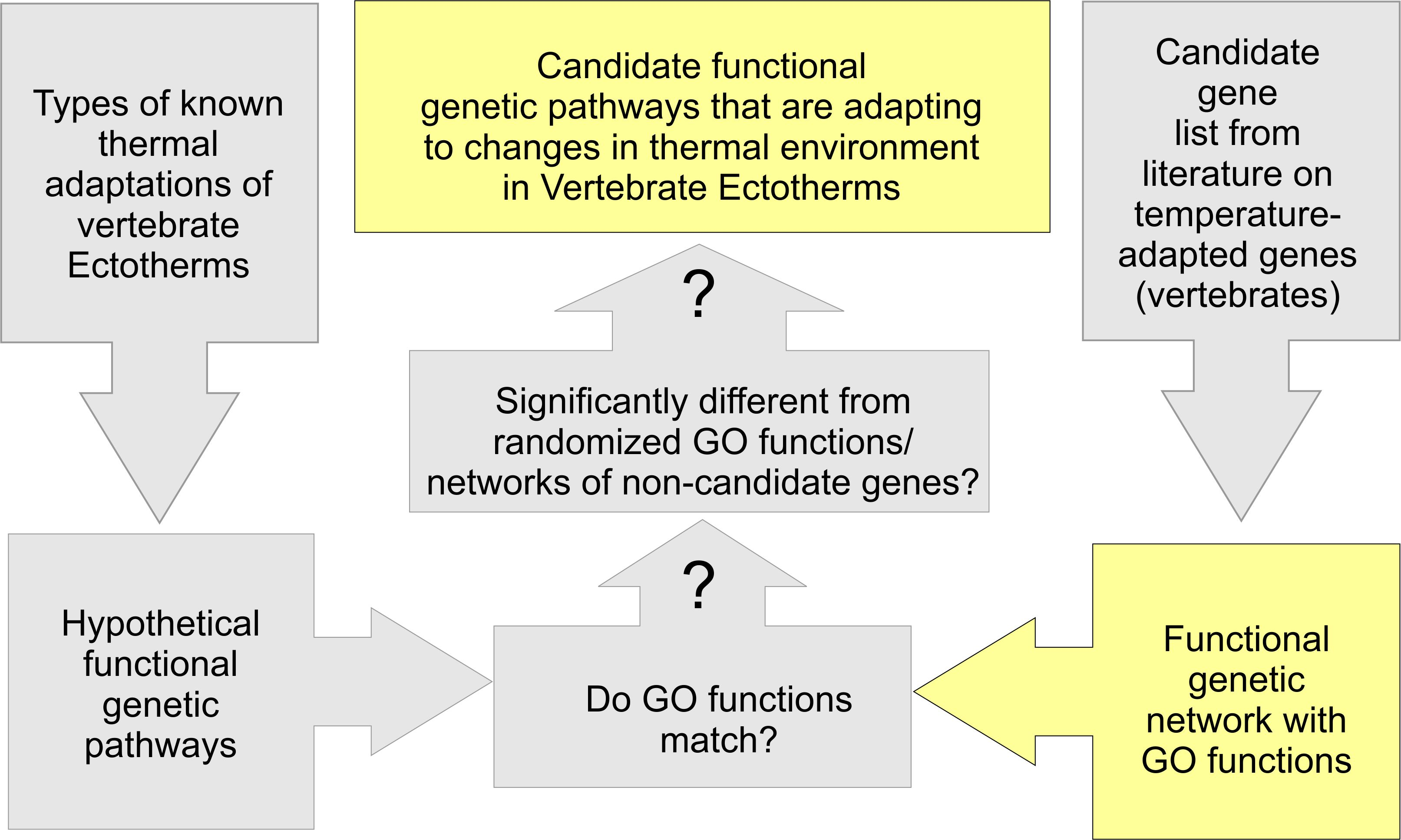


**Candidate Genes for thermal adaptation:** Genes that have been found to be adaptive (either by sequence modification, or by changes in expression levels) related to changes in the thermal environment.

**Functional Genetic Pathways:** All DNA segments in an organism that directly or indirectly interact with each other to perform a cellular function.

**Genetically overrepresented (GO) functions:** Abbreviation for Genetically Overrepresented Gene Ontology, which describes which functions are common in a genetic regulatory network. This includes the functions performed by single genes, as well as functions performed by interacting genes.

**Vertebrate Ectotherms:** Fish, Amphibians, and Reptiles.
