# Supplementary material for: A candidate multimodal functional genetic network for thermal adaptation
Source: PeerJ. 2014 Sep 30;2:e578. doi: 10.7717/peerj.578 (PMC4183952; doi:10.7717/peerj.578)
Supplement: Table S1 — Gene descriptions for humans were obtained from RefSeq via genecards.org [file peerj-02-578-s003.docx]

| **Locus in *Homo sapiens*** | **Name** | **Description of function in humans** (provided by RefSeq) | **Locus in *Gallus gallus*** | **Locus in *Anolis carolinensis*** |
| --- | --- | --- | --- | --- |
| **ADORA1** | Adenosine receptor A1 | Codes for an adenosine receptor that belongs to the G-protein coupled receptor 1 family. There are 3 types of adenosine receptors, each with a specific pattern of ligand binding and tissue distribution, and together they regulate a diverse set of physiologic functions. The type A1 receptors inhibit adenylyl cyclase, and play a role in the fertilization process. Animal studies also suggest a role for A1 receptors in kidney function and ethanol intoxication. Transcript variants with alternative splicing in the 5’ UTR have been found for this gene. | ADORA1 | Duplicated  ADORA1 |
| **ADORA2A** | Adenosine receptor 2A | This protein, an adenosine receptor of A2A subtype, uses adenosine as the preferred endogenous agonist and preferentially interacts with the G(s) and G (olf) family of G proteins to increase intracellular cAMP levels. It plays an important role in many biological functions, such as cardiac rhythm and circulation, cerebral and renal blood flow, immune function, pain regulation, and sleep. It has been implicated in pathophysiological conditions such as inflammatory diseases and neurodegenerative disorders. Alternative splicing results in multiple transcript variants. A read-through transcript composed of the upstream SPECC1L (sperm antigen with calponin homology and coiled-coil domains 1-like) and ADORA2A (adenosine A2a receptor) gene sequence has been identified, but it is thought to be non-coding. | ADORA2A | Duplicated  ADORA2A |
| **ADORA2B** | Adenosine receptor 2B | This gene encodes an adenosine receptor that is a member of the G protein-coupled receptor superfamily. This integral membrane protein stimulates adenylate cyclase activity in the presence of adenosine. This protein also interacts with netrin-1, which is involved in axon elongation. | ADORA2B | Not annotated. Possible ortholog |
| **ADORA3** | Adenosine receptor 3 | The receptor encoded by this gene mediates a sustained cardioprotective function during cardiac ischemia, it is involved in the inhibition of neutrophil degranulation in neutrophil-mediated tissue injury, it has been implicated in both neuroprotective and neurodegenerative effects, and it may also mediate both cell proliferation and cell death. Multiple transcript variants encoding different isoforms have been found for this gene | ADORA3 | N/A |
| **ADRB2** | Beta-2 adrenergic receptor, Surface | This gene encodes beta-2-adrenergic receptor which is a member of the G protein-coupled receptor superfamily. This receptor is directly associated with one of its ultimate effectors, the class C L-type calcium channel Ca(V)1.2. This receptor-channel complex also contains a G protein, an adenylyl cyclase, cAMP-dependent kinase, and the counterbalancing phosphatase, PP2A. The assembly of the signaling complex provides a mechanism that ensures specific and rapid signaling by this G protein-coupled receptor. This gene is intronless. Different polymorphic forms, point mutations, and/or downregulation of this gene are associated with nocturnal asthma, obesity and type 2 diabetes. | ADRB2 | ADRB2 adrenoceptor beta 2, surface |
| **AHSG** | alpha-2-HS-glycoprotein  FETUA protein | Alpha2-HS glycoprotein (AHSG), a glycoprotein present in the serum, is synthesized by hepatocytes. The AHSG molecule consists of two polypeptide chains, which are both cleaved from a proprotein encoded from a single mRNA. It is involved in several functions, such as endocytosis, brain development and the formation of bone tissue. The protein is commonly present in the cortical plate of the immature cerebral cortex and bone marrow hemopoietic matrix, and it has therefore been postulated that it participates in the development of the tissues. However, its exact significance is still obscure. | AHSG | AHSG |
| **AQP3** | Water transport channel | This gene encodes the water channel protein aquaporin 3. Aquaporins are a family of small integral membrane proteins related to the major intrinsic protein, also known as aquaporin 0. Aquaporin 3 is localized at the basal lateral membranes of collecting duct cells in the kidney. In addition to its water channel function, aquaporin 3 has been found to facilitate the transport of nonionic small solutes such as urea and glycerol, but to a smaller degree. It has been suggested that water channels can be functionally heterogeneous and possess water and solute permeation mechanisms | AQP3 | AQP3 |
| **AQP5** | Water transport channel | Aquaporin 5 (AQP5) is a water channel protein. Aquaporins are a family of small integral membrane proteins related  to the major intrinsic protein (MIP or AQP0). Aquaporin 5 plays a role in the generation of saliva, tears and  pulmonary secretions. AQP0, AQP2, AQP5, and AQP6 are closely related and all map to 12q13. | AQP5 | Four copies,not annotated yet |
| **AQP7** | Water transport channel | Aquaporins/major intrinsic protein (MIP) are a family of water-selective membrane channels. Aquaporin 7 has greater sequence similarity with AQP3 and AQP9 and they may be a subfamily. Aquaporin 7 and AQP3 are at the same chromosomal location suggesting that 9p13 may be a site of an aquaporin cluster. | AQP7 | Duplicated, not annotated yet |
| **CD36** | Thrombospondin Receptor | The protein encoded by this gene is the fourth major glycoprotein of the platelet surface and serves as a receptor for thrombospondin in platelets and various cell lines. Since thrombospondins are widely distributed proteins involved in a variety of adhesive processes, this protein may have important functions as a cell adhesion molecule. It binds to collagen, thrombospondin, anionic phospholipids and oxidized LDL. It directly mediates cytoadherence of Plasmodium falciparum parasitized erythrocytes and it binds long chain fatty acids and may function in the transport and/or as a regulator of fatty acid transport. Mutations in this gene cause platelet glycoprotein deficiency. Multiple alternatively spliced transcript variants encoding the same protein have been found for this gene. | CD36 | CD36 |
| **CETP** | Cholesteryl Ester Transfer Protein, Plasma | Cholesteryl ester transfer protein (CETP) transfers cholesteryl esters between lipoproteins. CETP may effect  susceptibility to atherosclerosis. among its related super-pathways are Lipoprotein metabolism and Metabolic pathways. GO annotations related to this gene include lipid binding and lipid transporter activity | CETP | CETP |
| **DSCR1** | Down Syndrome Chromosome Region | The protein encoded by this gene interacts with calcineurin A and inhibits calcineurin-dependent signaling pathways of genetic transcription, possibly affecting central nervous system development. Three transcript variants encoding three different isoforms have been found for this gene. In endothelial cells, VEGF stimulates RCAN1.4 expression which regulates gene expression, cell migration and tubular morphogenesis | -- | -- |
| **EDN1** | Endothelin 1  vasoconstrictor | The protein encoded by this gene is proteolytically processed to release a secreted peptide termed endothelin 1. This peptide is a potent vasoconstrictor and is produced by vascular endothelial cells. Endothelin 1 also can affect the central nervous system. Two transcript variants encoding different isoforms have been found for this gene. | EDN1 | EDN1 |
| **EGLN1** | Egl-9 Family Hypoxia-Inducible Factor 1 | The protein encoded by this gene catalyzes the post-translational formation of 4-hydroxyproline in hypoxia-inducible factor (HIF) alpha proteins. HIF is a transcriptional complex that plays a central role in mammalian oxygen homeostasis. This protein functions as a cellular oxygen sensor, and under normal oxygen concentration, modification by prolyl hydroxylation is a key regulatory event that targets HIF subunits for proteasomal destruction via the von Hippel-Lindau ubiquitylation complex. Mutations in this gene are associated with erythrocytosis familial type 3 (ECYT3). | LOC768374 | EGLN1 |
| **EGFR** |  | The protein encoded by this gene is a transmembrane glycoprotein that is a member of the protein kinase superfamily. This protein is a receptor for members of the epidermal growth factor family. EGFR is a cell surface protein that binds to epidermal growth factor. Binding of the protein to a ligand induces receptor dimerization and tyrosine autophosphorylation and leads to cell proliferation. Mutations in this gene are associated with lung cancer. | EGFR | EGFR |
| **FABP2** | Fatty Acid Binding Protein 2, Intestinal | The intracellular fatty acid-binding proteins (FABPs) belong to a multigene family with nearly twenty identified members. FABPs are divided into at least three distinct types, namely the hepatic-, intestinal- and cardiac-type. They form 14-15 kDa proteins and are thought to participate in the uptake, intracellular metabolism and/or transport of long-chain fatty acids. They may also be responsible in the modulation of cell growth and proliferation. Intestinal fatty acid-binding protein 2 gene contains four exons and is an abundant cytosolic protein in small intestine epithelial cells. This gene has a polymorphism at codon 54 that identified an alanine-encoding allele and a threonine-encoding allele. Thr-54 protein is associated with increased fat oxidation and insulin resistance. | FABP2 | FABP2 |
| **FABP3** | Fatty Acid Binding Protein 3, Muscle And Heart | The intracellular fatty acid-binding proteins (FABPs) belongs to a multigene family. FABPs are divided into at least three distinct types, namely the hepatic-, intestinal- and cardiac-type. They form 14-15 kDa proteins and are thought to participate in the uptake, intracellular metabolism and/or transport of long-chain fatty acids. They may also be responsible in the modulation of cell growth and proliferation. Fatty acid-binding protein 3 gene contains four exons and its function is to arrest growth of mammary epithelial cells. This gene is a candidate tumor suppressor gene for human breast cancer. |  |  |
| **HPSE** | Heparanase | Heparan sulfate proteoglycans are major components of the basement membrane and extracellular matrix. The protein encoded by this gene is an enzyme that cleaves heparin sulfate proteoglycans to permit cell movement through remodeling of the extracellular matrix. In addition, this cleavage can release bioactive molecules from the extracellular matrix. Several transcript variants encoding different isoforms have been found for this gene. | HPSE | HPSE |
| **HSF1** | Heat Shock Transcription Factor 1 | The product of this gene is a heat-shock transcription factor. Transcription of heat-shock genes is rapidly induced after temperature stress. Hsp90, by itself and/or associated with multichaperone complexes, is a major repressor of this gene. | HSF1 | HSF1 |
| **SERPINH1**  **HSP47** | Serpin Peptidase Inhibitor | Clade Heat shock protein 47. This gene encodes a member of the serpin superfamily of serine proteinase inhibitors. The encoded protein is localized to the endoplasmic reticulum and plays a role in collagen biosynthesis as a collagen-specific molecular chaperone. Autoantibodies to the encoded protein have been found in patients with rheumatoid arthritis. Expression of this gene may be a marker for cancer, and nucleotide polymorphisms in this gene may be associated with preterm birth caused by preterm premature rupture of membranes. | SERPINH1 | SERPINH1 |
| **HSPA14** | Heat shock 70kDa protein 14 | HSPA14 (heat shock 70kDa protein 14) is a protein-coding gene. Diseases associated with HSPA14 include anoxia, and endometrial carcinoma, and among its related super-pathways are Proteolysis Role of Parkin in the Ubiquitin-Proteasomal Pathway and Mechanisms of CFTR activation by S-nitrosoglutathione (normal and CF). GO annotations related to this gene include protein binding and ATP binding | HSPA14 | HSPA14 |
| **HSPA4** | Heat Shock 70kDa Protein 4 | HSPA4 (heat shock 70kDa protein 4) is a protein-coding gene. Diseases associated with HSPA4 include autoimmune inner ear disease, and chronic cervicitis, and among its related super-pathways are Proteolysis Role of Parkin in the Ubiquitin-Proteasomal Pathway and Mechanisms of CFTR activation by S-nitrosoglutathione (normal and CF). GO annotations related to this gene include ATP binding. An important paralog of this gene is HYOU1. | HSPA4 | HSPA4 |
| **HSPA4L** | Heat Shock 70kDa Protein 4-like | HSPA4L (heat shock 70kDa protein 4-like) is a protein-coding gene. Diseases associated with HSPA4L include endolymphatic hydrops, and hypertension. GO annotations related to this gene include ATP binding. An important paralog of this gene is HYOU1 | HSPA4L | HSPA4L |
| **HSPA8** | Heat Shock 70kDa Protein 8 | This gene encodes a member of the heat shock protein 70 family, which contains both heat-inducible and constitutively expressed members. This protein belongs to the latter group, which are also referred to as heat-shock cognate proteins. It functions as a chaperone, and binds to nascent polypeptides to facilitate correct folding. It also functions as an ATPase in the disassembly of clathrin-coated vesicles during transport of membrane components through the cell. | HSPA8 | Duplicated  HSPA8 |
| **HSPB2** | Heat Shock 27kDa Protein 2 | The protein encoded by this gene belongs to the superfamily of small heat-shock proteins containing a conservative alpha-crystallin domain at the C-terminal part of the molecule. The protein is expressed preferentially in the heart and skeletal muscle. This protein regulates Myotonic Dystrophy Protein Kinase, which plays an important role in maintenance of muscle structure and function | HSPB2 | Not annotated |
| **HSPH1** | Heat Shock 105kDa/110kDa Protein 1 | The protein encoded by this gene belongs to the superfamily of small heat-shock proteins containing a conservative  alpha-crystallin domain at the C-terminal part of the molecule. The protein is expressed preferentially in the heart and skeletal muscle. This protein regulates Myotonic Dystrophy Protein Kinase, which plays an important role in maintenance of muscle structure and function. | HSPB2 | HSPB2 |
| **LEPR** | Leptin Receptor | The protein encoded by this gene belongs to the gp130 family of cytokine receptors that are known to stimulate gene transcription via activation of cytosolic STAT proteins. This protein is a receptor for leptin (an adipocyte-specific hormone that regulates body weight), and is involved in the regulation of fat metabolism, as well as in a novel hematopoietic pathway that is required for normal lymphopoiesis. Mutations in this gene have been associated with obesity and pituitary dysfunction. Alternatively spliced transcript variants encoding different isoforms have been described for this gene. It is noteworthy that this gene and LEPROT gene (GeneID:54741) share the same promoter and the first 2 exons, however, encode distinct proteins | LEPR | Duplicated  LEPR |
| **LPL** | Lipoprotein Lipase | LPL encodes lipoprotein lipase, which is expressed in heart, muscle, and adipose tissue. LPL functions as a homodimer, and has the dual functions of triglyceride hydrolase and ligand/bridging factor for receptor-mediated lipoprotein uptake. Severe mutations that cause LPL deficiency result in type I hyperlipoproteinemia, while less extreme mutations in LPL are linked to many disorders of lipoprotein metabolism. | LPL | LPL |
| **LPA**  **PLG** | Lipoprotein, Lp(A) | The protein encoded by this gene is a serine proteinase that inhibits the activity of tissue-type plasminogen activator I. The encoded protein constitutes a substantial portion of lipoprotein(a) and is proteolytically cleaved, resulting in fragments that attach to atherosclerotic lesions and promote thrombogenesis. Elevated plasma levels of this protein are linked to atherosclerosis. Depending on the individual, the encoded protein contains 2-43 copies of kringle-type domains. The allele represented here contains 15 copies of the kringle-type repeats and corresponds to that found in the reference genome sequence | PLG | Not named yet |
| **MAPK1** | Mitogen-Activated Protein Kinase 1 | The protein encoded by this gene is a member of the MAP kinase family. MAP kinases, also known as extracellular signal-regulated kinases (ERKs), act as an integration point for multiple biochemical signals, and are involved in a wide variety of cellular processes such as proliferation, differentiation, transcription regulation and development. The activation of this kinase requires its phosphorylation by upstream kinases. Upon activation, this kinase translocates to the nucleus of the stimulated cells, where it phosphorylates nuclear targets | MAPK1 | MAPK1 |
| **MAPK14** | Mitogen-Activated Protein Kinase 14 | This kinase is activated by various environmental stresses and proinflammatory cytokines. The activation requires its phosphorylation by MAP kinase kinases (MKKs), or its autophosphorylation triggered by the interaction of MAP3K7IP1/TAB1 protein with this kinase. The substrates of this kinase include transcription regulator ATF2, MEF2C, and MAX, cell cycle regulator CDC25B, and tumor suppressor p53, which suggest the roles of this kinase in stress related transcription and cell cycle regulation, as well as in genotoxic stress response. | MAPK14 | MAPK14 |
| **Mc1R** | Melanocortin 1 Receptor (Alpha Melanocyte Stimulating Hormone Receptor) | This intronless gene encodes the receptor protein for melanocyte-stimulating hormone (MSH). The encoded protein, a seven pass transmembrane G protein coupled receptor, controls melanogenesis. Two types of melanin exist: red pheomelanin and black eumelanin. Gene mutations that lead to a loss in function are associated with increased pheomelanin production, which leads to lighter skin and hair color. Eumelanin is photoprotective but pheomelanin may contribute to UV-induced skin damage by generating free radicals upon UV radiation. Binding of MSH to its receptor activates the receptor and stimulates eumelanin synthesis. This receptor is a major determining factor in sun sensitivity and is a genetic risk factor for melanoma and non-melanoma skin cancer. Over 30 variant alleles have been identified which correlate with skin and hair color, providing evidence that this gene is an important component in determining normal human pigment variation | MC1R | Not in the database |
| **MC2R** |  | MC2R is selectively activated by adrenocorticotropic hormone. Mutations in MC2R can result in familial glucocorticoid deficiency. | MC2R | MC2R |
| **MC4R** |  | The protein encoded by this gene is a membrane-bound receptor and member of the melanocortin receptor family. The encoded protein interacts with adrenocorticotropic and MSH hormones and is mediated by G proteins. This is an intronless gene. Defects in this gene are a cause of autosomal dominant obesity. | MC4R | MC4R |
| **MMRN1** | Multimerin 1 | Multimerin is a massive, soluble protein found in platelets and in the endothelium of blood vessels. It is comprised of subunits linked by interchain disulfide bonds to form large, variably sized homomultimers. Multimerin is a factor V/Va-binding protein and may function as a carrier protein for platelet factor V. It may also have functions as an extracellular matrix or adhesive protein. Recently, patients with an unusual autosomal-dominant bleeding disorder (factor V Quebec) were found to have a deficiency of platelet multimerin. | MMRN1 | MMRN1 |
| **MRAS** | Muscle RAS Oncogene Homolog | a member of the Ras family of small GTPases. These membrane-associated proteins function as signal transducers in multiple processes including cell growth and differentiation, and dysregulation of Ras signaling has been associated with many types of cancer. | MRAS | MRAS |
| **MT-ATP6** | Mitochondrially Encoded ATP Synthase 6 | Mitochondrial membrane ATP synthase (F(1)F(0) ATP synthase or Complex V) produces ATP from ADP in the presence of a proton gradient across the membrane which is generated by electron transport complexes of the respiratory chain. | ATP6 | ATP6 |
| **PSIP2 LEDGF** | PC4 And SFRS1 Interacting Protein 1 | Transcriptional coactivator involved in neuroepithelial stem cell differentiation and neurogenesis.Involved in particular in lens epithelial cell gene regulation and stress responses. May play an important role in lens epithelial to fiber cell terminal differentiation. May play a protective role during stress-induced apoptosis | PSIP1 | PSIP1 |
| **POMC** | Proopiomelanocortin | This gene encodes a polypeptide hormone precursor that undergoes extensive, tissue-specific, post-translational processing via cleavage by subtilisin-like enzymes known as prohormone convertases. The encoded protein is synthesized mainly in corticotroph cells of the anterior pituitary where four cleavage sites are used; adrenocorticotrophin, essential for normal steroidogenesis and the maintenance of normal adrenal weight, and lipotropin beta are the major end products. In other tissues, including the hypothalamus, placenta, and epithelium, all cleavage sites may be used, giving rise to peptides with roles in pain and energy homeostasis, melanocyte stimulation, and immune modulation. These include several distinct melanotropins, lipotropins, and endorphins that are contained within the adrenocorticotrophin and beta-lipotropin peptides. Mutations in this gene have been associated with early onset obesity, adrenal insufficiency, and red hair pigmentation | POMC | POMC |
| **RAPTOR**  **RPTOR** | Regulatory Associated Protein Of MTOR, Complex 1 | This gene encodes a component of a signaling pathway that regulates cell growth in response to nutrient and insulin levels. The encoded protein forms a stoichiometric complex with the mTOR kinase, and also associates with eukaryotic initiation factor 4E-binding protein-1 and ribosomal protein S6 kinase. The protein positively regulates the downstream effector ribosomal protein S6 kinase, and negatively regulates the mTOR kinase. Multiple transcript variants encoding different isoforms have been found for this gene. | RPTOR | Duplicated, not annotated |
| **SOD1** | Superoxide Dismutase 1, Soluble | The protein encoded by this gene binds copper and zinc ions and is one of two isozymes responsible for destroying free superoxide radicals in the body. The encoded isozyme is a soluble cytoplasmic protein, acting as a homodimer to convert naturally-occuring but harmful superoxide radicals to molecular oxygen and hydrogen peroxide. The other isozyme is a mitochondrial protein. Mutations in this gene have been implicated as causes of familial amyotrophic lateral sclerosis. | SOD1 | SOD1 |
| **UNG** | uracil-DNA glycosylase | This gene encodes one of several uracil-DNA glycosylases. One important function of uracil-DNA glycosylases is to prevent mutagenesis by eliminating uracil from DNA molecules by cleaving the N-glycosylic bond and initiating the base-excision repair (BER) pathway. Uracil bases occur from cytosine deamination or misincorporation of dUMP residues. Alternative promoter usage and splicing of this gene leads to two different isoforms: the mitochondrial UNG1 and the nuclear UNG2. The UNG2 term was used as a previous symbol for the CCNO gene (GeneID 10309), which has been confused with this gene, in the literature and some databases. | UNG | N/A  Possibly SMUG1 |
| **UCP1**  **UCP2**  **UCP3** | Uncoupling Protein 1 (Mitochondrial, Proton Carrier) | Mitochondrial uncoupling proteins (UCP) are members of the family of mitochondrial anion carrier proteins (MACP). UCPs separate oxidative phosphorylation from ATP synthesis with energy dissipated as heat, also referred to as the mitochondrial proton leak. UCPs facilitate the transfer of anions from the inner to the outer mitochondrial membrane and the return transfer of protons from the outer to the inner mitochondrial membrane. This gene is expressed only in brown adipose tissue, a specialized tissue which functions to produce heat | UCP3 | UCP3 |
